# Thrombin generation, bleeding and hemostasis in humans: Protocol for a scoping review of the literature

**DOI:** 10.1371/journal.pone.0293632

**Published:** 2023-11-01

**Authors:** Joseph R. Shaw, Tyler James, Jonathan Douxfils, Yesim Dargaud, Jerrold H. Levy, Herm Jan M. Brinkman, Risa Shorr, Deborah Siegal, Lana A. Castellucci, Peter Gross, Roy Khalife, Christine Sperling, David Page, Dean Fergusson, Marc Carrier

**Affiliations:** 1 Department of Medicine, University of Ottawa and the Ottawa Hospital Research Institute, Ottawa, Canada; 2 The Ottawa Hospital, Ottawa, Canada; 3 Namur Thrombosis and Hemostasis Center, University of Namur, Namur, Belgium; 4 Lyon Hemophilia Center and Clinical Haemostasis Unit, Lyon, France; 5 Department of Anesthesiology, Duke University School of Medicine, Durham, North Carolina, United States of America; 6 Department of Molecular Hematology, Sanquin Research, Amsterdam, The Netherlands; 7 Department of Medicine, Division of Hematology and Thromboembolism, McMaster University, Hamilton, Canada; 8 CanVECTOR Patient Partner, Ottawa, Canada; 9 Canadian Hemophilia Society Patient Partner, Montreal, Canada; University of Ferrara, ITALY

## Abstract

**Introduction:**

Hemostasis and bleeding are difficult to measure. Thrombin generation assays (TGAs) can measure both procoagulant and anticoagulant contributions to coagulation. TGAs might prove useful for the study of bleeding disorders. There has been much progress in TGA methodology over the past two decades, but its clinical significance is uncertain. We will undertake a scoping review of the literature to synthesize available information on the application of TGAs towards the study of bleeding and hemostasis, TGA methodologies being used and to summarize available literature on associations between TGA parameters, bleeding and hemostatic outcomes.

**Methods and analysis:**

MEDLINE, EMBASE and the Cochrane Central Register of Controlled Trials (CENTRAL) will be searched in collaboration with an information specialist. Title/abstract and full-text screening will be carried out independently and in duplicate; eligible study types will include randomized controlled trials, non-randomized studies, systematic reviews, and case series reporting TGA results and bleeding/hemostatic outcomes among humans. Mapping the information identified will be carried out with results presented using qualitative data analytical techniques.

**Ethics and dissemination:**

This scoping review will use published, publicly available information. Research ethics approval will not be required. We will disseminate our findings using conference presentations, peer-reviewed publications, social media, and engagement with knowledge users. This review will outline knowledge gaps concerning TGAs, better delineate its applicability as a clinically relevant assay for bleeding. and seek to identify ongoing barriers to its widespread adoption in clinical research, and eventually, in the clinical setting.

**Trail regulations:**

**Registration ID with Open Science Framework:** osf.io/zp4ge.

## Introduction

### Why measure thrombin generation?

Thrombin plays a central role in the process of coagulation. It is involved in the amplification of coagulation and the conversion of fibrinogen to fibrin, leading to the formation of a fibrin clot. Moreover, thrombin contributes to feedback amplification of coagulation, platelet activation, as well as the regulation of coagulation through its thrombomodulin-mediated effects on the activated protein C pathway (APC), regulation of the fibrinolytic system (e.g., thrombin-activatable fibrinolysis inhibitor; TAFI) and its direct influence on clot structure [1,2]. Ultimately, the coagulability of blood is dictated by its potential to generate thrombin and the resulting downstream effects of thrombin on coagulation, anticoagulation, and fibrinolytic pathways. Besides its essential role in hemostasis, thrombin also elicits a host of responses in the vascular endothelium, including shape and permeability changes, mobilization of adhesive molecules to the endothelial surface and stimulation of cytokine production [3]. Measuring thrombin directly provides an evaluation of the total enzymatic work carried out by the coagulation cascade [4]. Coagulation can be affected by genetic factors, disease states, endogenous inhibitors of coagulation, exogenous hormonal exposures, and anticoagulation therapies. By measuring this final step of the coagulation cascade, thrombin generation testing provides a simple approach that, in theory, accounts for both procoagulant and anticoagulant factors. In this respect, it has been referred to as a “global” coagulation assay. Quantifying thrombin, given its pivotal role in coagulation, might provide a comprehensive measure of the coagulation phenotype [5].

### An overview of thrombin generation assays (TGAs)

**Fig 1:**
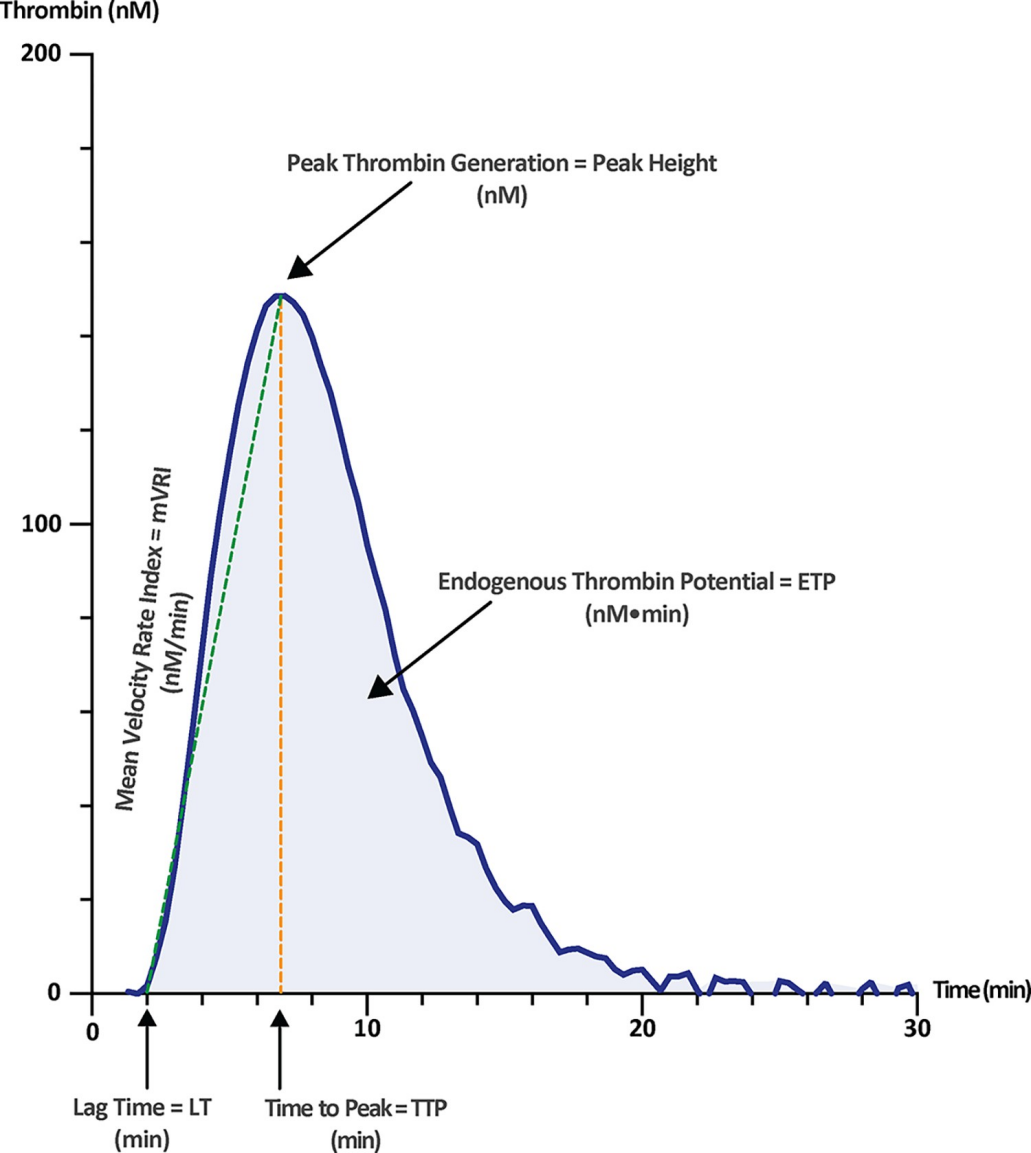
The thrombogram. Components of a typical thrombin generation curve generated using the Calibrated Automated Thrombography (CAT) assay. LT = lag time; TTP = time to peak; ETP = endogenous thrombin potential; mVRI = mean velocity rate index (calculated as peak height/[TTP-LT]).

McFarlane and Biggs first introduced thrombin generation testing in the 1950s [6]. This first assay involved manual subsampling of unaltered whole blood and measuring of fibrinogen clotting times, with results compared to a thrombin-dilution calibration curve. This approach was laborious, time consuming, and provided a limited picture of thrombin generation due to the small number of data points collected during the procedure. Hemker and colleagues later made substantial changes to the thrombin generation assay in 1993 using defibrinated plasma, a slow-reacting chromogenic substrate, and continuous registration of thrombin activity [7]. The implementation of a fluorogenic substrate permitted the measurement of thrombin generation in fibrinogen-containing media, including both citrated platelet-poor plasma (PPP) and platelet-rich plasma (PRP) [8]. The evolution of calibration methodology, in concert with the use of a continuous plate fluorescence reader, laid the groundwork for a modern iteration of the TGA, known as Calibrated Automated Thrombography (CAT) [9]. TGAs provide information on the kinetic and quantitative parameters of thrombin generation (**Fig 1**) [10]. Kinetic parameters include the lag time (LT; time for thrombin generation to first occur in minutes) and the time to peak (TTP; start to maximal thrombin generation in minutes), whereas quantitative measures include the peak height (“peak”; maximal thrombin generation, in nanomolar; nM) and the endogenous thrombin potential (ETP; area under the thrombin generation curve, in nM•min). The velocity index is a composite kinetic/quantitative parameter commonly derived by dividing peak height by the difference between TTP and LT (i.e., [peak/(TTP-LT)], referred to as the mean velocity rate index; mVRI in nM/min).

There are 5 commercially available automated TGA systems: CAT/Thrombinoscope^®^ (Diagnostica Stago), ST-Genesia^®^ (Diagnostica Stago), Innovance ETP^®^ (Siemens Healthcare), Technothrombin^®^ (Technoclone) and Ceveron^®^ TGA (Technoclone). CAT/Thrombinoscope, ST-Genesia, Technothrombin and Ceveron TGA use fluorogenic analysis methods, whereas Innovance ETP is a chromogenic assay. Calibration is achieved using a thrombin-based calibrator with known thrombin activity. This is done using either human thrombin when calibration is carried out in buffer solution or using a complex of α-2-macroglobulin and thrombin when calibration is carried out in clotting plasma. Free thrombin cannot be used as a calibrator in clotting plasma due to the presence of antithrombins, whereas α-2-macroglobulin-thrombin complex is not affected by antithrombins. Thrombin generation is usually triggered using tissue factor (TF) with concentrations ranging from 0–20 pM for the fluorogenic assays, depending on the question under study. Higher TF concentrations are typically used for chromogenic assays [11]. With increasing TF concentration, a trade-off occurs between experimental variability and the determinants of thrombin generation. Experimental variability is reduced with higher TF concentrations, but sensitivity towards intrinsic pathway coagulation factors is lower (i.e., factors VIII, IX and XI), to the point of being negligible at TF concentrations of 5 pM or greater. Corn trypsin inhibitor can be added to sample collection tubes to mitigate the impact of contact activation, which appears to be mainly relevant when using TF concentrations of 5 pM or lower [12]. The thrombin generation assay can also be modified by adding thrombomodulin (TM) when studying the APC pathway [10,13].

With greater complexity, sensitivity, and analytical versatility comes greater susceptibility to preanalytical variables. Throughout its first decade of use, substantial heterogeneity was observed using CAT methodology based on the assay reagents, experimental conditions, and analytical techniques. Several preanalytical variables can also affect TGA measurements, including blood collection/phlebotomy techniques, type of collection tube, the presence of an anticoagulant (e.g., heparin), use of corn trypsin inhibitor, centrifugation techniques [14], and sample storage/freezing [13,15,16]. Differences in preanalytical and analytical conditions produced significant interlaboratory variability, despite low intra-assay/inter-assay coefficient of variation (CV) values and consistent intraindividual TGA measurements [4,17]. This variability of analytic conditions has hampered clinical enthusiasm for applying TGAs to study thrombotic or hemorrhagic conditions over the past two decades.

Increasing recognition and mitigation of preanalytical considerations has enhanced the interpretation and potential clinical applicability of TGAs [12,14,18,19]. Progress has been made in terms of standardization [20–22], the development of commercially available standard TF trigger reagents, and the publication of ISTH SSC communications on recommended TGA assay methodology [23,24]. Analyses using ≥ 5 pM TF concentrations seem less prone to preanalytical variation [12]. A recent review of the literature identified increasing consistency with TGA reaction conditions over the past decade when used to study direct oral anticoagulants (DOACs) and reversal [25]. This progress could instill growing confidence in the interpretation and clinical relevance of TGA results. Moreover, manufacturers have recently attempted to produce fully automated TGA analyzers, such as the ST-Genesia platform, developed for clinical applications [16,26]. This new analyzer uses a set of trigger reagents with varying TF concentrations (STG-BleedScreen; STG-ThromboScreen; STG-DrugScreen), depending on the clinical question being investigated, in addition to novel calibration methodology, quality control samples, and normalization of results using a standard reference plasma.

The best approaches for reporting and analyzing thrombin generation data are unclear. TGA parameters are drawn from the thrombin generation curve. These parameters describe changes to what is, fundamentally, a complex measure (i.e., the thrombin generation curve itself). However, this can lead to an oversimplification of the changes observed in disease states or induced by treatments such as anticoagulants and hemostatic therapies. Further, the interdependence of TGA parameters can lead to challenges when statistical methodologies are applied indiscriminately, without carefully considering the relationship between parameters (e.g., collinearity in regression models). Lastly, selective reporting of specific TGA parameters (e.g., the ETP) can lead to reporting bias and provide an incomplete picture of the data.

### Conventional coagulation assays versus thrombin generation assays

Conventional coagulation assays, such as the prothrombin time (PT) and activated partial thromboplastin time (aPTT), are used to measure the procoagulant activity of the extrinsic and intrinsic pathways of coagulation, respectively. Coagulation in vivo occurs through more complex, concurrent, and dynamic interactions within a network of enzymatic components than reflected by these pathways. Conventional coagulation assays are conceptually analogous to the TGA lag time, but instead use clot formation as the endpoint of interest. Conversion of fibrinogen to fibrin occurs when less than 5% of total thrombin within a sample has been generated, meaning that conventional clot-based assays fail to capture most of the information available pertaining to in vivo coagulation processes [4]. They are unable to detect hypercoagulability in the setting of congenital or acquired thrombophilia [27]. Further, conventional coagulation assays largely fail to capture the effects of DOACs on coagulation. Conventional clotting assays demonstrate a low sensitivity to what may be clinically significant DOAC concentrations, and reagent dependent performance for both sensitivity and correlation with DOAC levels [28,29].

TGAs can detect changes to the coagulation system that are not otherwise captured using conventional clotting assays [30]. For example, measurement of thrombin generation has been instrumental in challenging the belief that patients with cirrhosis and INR elevation are coagulopathic and has contributed to the theory of rebalanced hemostasis in liver disease [31,32]. In addition, patients with severe hemophilia but with a mild bleeding phenotype have higher ETP values as compared to those with a typical bleeding tendency [33]. Similarly, an analysis of patients with rare bleeding disorders found that kinetic TGA parameters were significantly associated with bleeding risk and showed a significant positive correlation with bleeding score values, whereas no association was found between the PT or aPTT and bleeding scores [34]. The administration of bypassing therapies such as activated prothrombin complex concentrates (aPCCs) or recombinant factor VIIa (rFVIIa) to patients with hemophilia and inhibitors leads to correction of both kinetic and quantitative parameters of thrombin generation [35], but has little effect on conventional coagulation assays such as the aPTT [36].

### Clinical hemostasis, bleeding, and thrombin generation as a surrogate outcome

Measurement of bleeding and hemostasis can be challenging [37]. In the case of patients with hemophilia, the absolute frequency of bleeding episodes facilitates detection of a significant effect from replacement or bypassing therapies (e.g., annualized bleed rates; ABRs) within individual patients [38]. In other cases, such as anticoagulant-associated bleeding, intraindividual bleed frequency is lower, but the absolute number of patients at risk of bleeding is much greater due to the prevalence of oral anticoagulant usage. In cases such as these, detecting an impact from hemostatic therapy or a specific reversal agent on hemostasis with respect to an individual bleeding event can be very difficult. Although clinical hemostasis remains the goal of anticoagulation reversal, hemostatic efficacy is very hard to measure. Clinical criteria have been developed for adjudicating hemostasis among studies of hemostatic or anticoagulation reversal therapies [39], but the construct validity of current definitions has never been formally evaluated.

Thrombin generation could serve as a useful surrogate outcome among studies measuring bleeding, whether secondary to clinical pathology (e.g., hemophilia) or due to antithrombotic therapy. Several studies have demonstrated the clinical relevance of TGA parameters for bleeding outcomes in patients with hemophilia [40,41], patients undergoing surgery [42], or anticoagulated patients [43,44]. Conversely, there remains significant uncertainty concerning the clinical significance of TGA results, and whether differences in TGA parameters necessarily translate to meaningful clinical differences. Further, the relative importance of kinetic and quantitative TGA parameters concerning bleeding and hemostasis is unknown.

### Objectives

We will conduct a scoping review to address the following objectives:

1. Provide a synthesis of the published literature on the use of thrombin generation assays for measuring bleeding and/or hemostasis.
2. Summarize the thrombin generation assay methodology being used to study bleeding and/or clinical hemostasis.
3. Summarize the literature on associations between thrombin generation parameters and bleeding/hemostatic outcomes.

## Study design

Scoping reviews are a suggested methodology for mapping literature around a particular topic. Scoping reviews are useful when appraising innovative technologies, particularly those that have not been comprehensively reviewed and exhibit a large, complex or heterogenous nature, not amenable to conventional systematic review methodology [45]. A scoping review maps existing sources of evidence and can be used to summarize and disseminate research findings through a structured approach. Based on the originality of the research technology being evaluated and the anticipated heterogenous nature of studies applying thrombin generation towards the measurement of bleeding and hemostasis, we chose to conduct a scoping review to map and synthesize the desired evidence-base. This scoping review will adhere to the framework proposed by Arksey and O’Malley and updated by Levac and colleagues [46,47], in addition to guidance published by the Joanna Briggs Institute [45,48].

### Research questions

This scoping review will address the following two research questions:

1. “What thrombin generation methodology, reagents and assay analytical/preanalytical conditions are being used to measure bleeding or hemostasis?”
2. “Are alterations in thrombin generation parameters correlated with bleeding and clinical hemostatic outcomes?”

### Protocol and registration

This protocol adheres to the Preferred Reporting Items for Systematic Reviews and Meta-Analyses extension for scoping reviews (PRISMA-ScR) [49] and has been registered with the Open Science Framework (osf.io/zp4ge). Given the iterative and reflexive nature of scoping reviews, amendments to the current protocol may be needed and will be reported transparently in the final study report [45].

### Eligibility criteria

As outlined by Arksey and O’Malley [46], we have a broad, flexible and iterative approach to both our search strategy and eligibility criteria to ensure comprehensive coverage of the published literature.

#### Clinical conditions

Clinical disorders of interest include congenital bleeding disorders (e.g., hemophilia, von Willebrand disease, rare coagulation factor deficiencies), patients with acquired bleeding disorders (e.g., immune thrombocytopenia, acquired inhibitors of coagulation), participants treated with antithrombotic therapy (antiplatelet agents, anticoagulation therapy), participants subjected to a hemostatic challenge (e.g., surgery, skin punch biopsy) or participants treated with hemostatic/anticoagulation reversal agents. Studies evaluating human volunteers or clinical study participants of any age, sex/gender, or race will be included. Studies exclusively including animal subjects will be excluded. Eligible bleeding disorders/disease states are outlined in **Table 1**, eligible antithrombotic therapies are outlined in **Table 2**, eligible hemostatic/reversal agents and eligible therapies for congenital/acquired bleeding disorders are outlined in **Table 3**. These lists are not exhaustive and will be updated during the conduct of the scoping review.

**Table 1:** Eligibility criteria–bleeding disorders and conditions.

	Bleeding Disorder	Diagnosis
**Congenital disorders of hemostasis**	Hemophilia A	---
	Hemophilia B	---
	Von Willebrand disease (VWD)	---
	Disorders of fibrinogen and fibrinolysis	Afibrinogenemia Dysfibrinogenemia Hypodisfibrinogenemia
	Platelet function defects	Bernard-Soulier syndrome Gray platelet syndrome May-Hegglin anomaly Storage pool disorders (Wiskott-Aldrich syndrome, Chediak-Higashi, Hermansky-Pudlak) Glanzmann thrombasthenia
	Rare bleeding disorders (RBDs)/ rare inherited coagulation disorders (RICDs)/Rare coagulation deficiencies (RCDs)	---
**Acquired disorders of hemostasis**	Antithrombotic therapy	see **Table 2**
	Acquired inhibitors of coagulation (“acquired hemophilia”)	---
	Consumptive coagulopathy/ Disseminated Intravascular Coagulation (DIC)	---
	Liver disease Cirrhosis	---
	Acquired disorders of platelet function	Uremic platelet dysfunction Immune thrombocytopenia (ITP) Platelet dysfunction secondary to cardiopulmonary bypass
	Thrombocytopenia	---
	Acquired von Willebrand syndrome (AVWS)	---
**Iatrogenic bleeding**	Surgical bleeding Procedural bleeding	---
	Human volunteer bleeding models	Skin punch biopsy

**Table 2:** Eligibility criteria–antithrombotic therapies.

	Drug Class	Medication
**Anticoagulants**	Vitamin K Antagonists	Warfarin
		Acenocouramol
		Phenprocoumon
		Fluindione
	Direct Factor Xa Inhibitors (FXaI)	Apixaban
		Betrixaban
		Edoxaban
		Rivaroxaban
	Direct Thrombin Inhibitors (DTI)	Dabigatran
		Melagatran/Ximelagatran
		Argatroban
		Bivalirudin
	Unfractionated Heparin (UFH)	
	Low Molecular Weight Heparins (LMWH)	Dalteparin
		Enoxaparin
		Nadroparin
		Semuloparin
		Tinzaparin
	Indirect Factor Xa Inhibitors	Fondaparinux
	Heparinoids	Danaparoid
	Factor XIa Inhibitors	IONIS-FXI_RX_
		Osocimab
		Abelacimab
		Milvexian
		Xisomab 3G3
		Fesomersen
		Asundexian
**Antiplatelet Agents**	Non-steroidal anti-inflammatory drug (NSAID)	Acetylsalicylic acid (ASA)/ Aspirin
	P2Y12 Inhibitors	Clopidogrel
		Prasugrel
		Ticagrelor
		Ticlopidine
		Cangrelor
	GPIIb/IIIa receptor antagonists	Abciximab
	cAMP phosphodiesterase inhibitors	Dipyridamole

**Table 3:** Eligible hemostatic agents and therapies for congenital/acquired bleeding disorders.

Therapy Class	Hemostatic Agent
**Prothrombin Complex Concentrate (PCC)**	3-factor PCC (e.g. Bebulin^®^)
	4-factor PCC (e.g. Octaplex^®^)
**Activated Prothrombin Complex Concentrate (aPCC)**	aPCC (e.g. FEIBA^®^)
**Recombinant Factor VIIa (rFVIIa)**	rFVIIa (e.g. Novoseven^®^, Sevenfact^®^)
**Fresh Frozen Plasma (FFP)**	---
Andexanet Alfa (Andexxa^®^/Ondexxya^®^)	---
Idarucizumab (Praxbind^®^)	---
**Ciraparantag (aripazine)**	---
**Antifibrinolytics**	Tranexamic Acid Aminocaproic Acid
**Fibrinogen Concentrates**	RiaSTAP
	Fibryga
	Clottafact^®^
**Factor VII Concentrates**	Pd-FVII
**Factor VIII Products**	Advate
	Hemofil M
	Kogenate FS
	Koate
	Kovaltry
	Novoeight
	Nuwiq
	Recombinate
	Xyntha
	Adynovate
	Afstyla
	Eloctate
	Esperoct
	Jivi
	Factane
	Octanate
	Efanesoctocog alfa (Altuviiio^®^)
**Recombinant Porcine Sequence Factor VIII Concentrate**	Obizur
**Factor IX Products**	AlphaNine SD
	BeneFIX
	Ixinity
	Mononine
	Rixubis
	Alprolix
	Idelvion
	Rebinyn
**Factor X Products**	Coagadex
**Factor XI Products**	Pd-FXI
	Hemoleven^®^
**Factor XIII Products**	Corifact
	Tretten
**Bispecific Monoclonal Antibody**	Emicizumab (ACE910; Hemlibra^®^)
**Anti-TFPI agents**	Concizumab
	Marstacimab
**RNA Interference Therapy**	Fitusiran
**Aptamers**	BT200
**Von Willebrand Concentrates**	Humate-P
	Alphanate
	Wilate
	Voncento
**Recombinant VWF**	Vonvendi

#### Concept

This scoping review’s focus and key concept will be how thrombin generation assays are applied to study bleeding disorders and clinical hemostasis. Studies will be considered if they measure thrombin generation and report clinical bleeding or hemostatic outcomes, as outlined below.

#### Thrombin generation assays and thrombin generation parameters

Thrombin generation assays considered are outlined in **Table 4**. Additional assays identified through our literature search will be considered, following discussion and agreement among study team members. To be considered for inclusion, thrombin generation must be measured using assays that fall within conventional definitions of a “thrombin generation test” or “thrombin generation assay” [9,27], using either chromogenic or fluorogenic substrates and reporting results as either relative fluorescent units (RFU) or calibrated thrombin units [11].

**Table 4:** Eligible thrombin generation assays.

Thrombin Generation Assay	Manufacturer
CAT/Thrombinoscope (Calibrated Automated Thrombography)	Diagnostica Stago
**ST-Genesia**	Diagnostica Stago
**Innovance ETP**	Siemens
**Technothrombin**	Technoclone
**Ceveron Alpha TGA**	Diapharma
Nijmegen Hemostasis Assay/ Novel Hemostasis Assay (NHA)	N/A (Academic; Radboud University Medical Center)

For this review, “thrombin generation parameters” will refer to the LT, TTP, peak thrombin generation, the ETP and the mVRI, as defined above (**Fig 1**). This terminology will be used consistently throughout the review. Studies describing these same parameters in terms of their measurement, but using different terminology (e.g., “maxIIa” for peak thrombin generation in nM) will be included, provided that the parameter is clearly defined in the study methods. Studies exclusively reporting indirect (occasionally referred to as “in vivo”) measures of thrombin generation such as prothrombin fragment 1+2 (F 1+2), thrombin-antithrombin complexes (TAT) or d-dimer measurements will be excluded, as these “in vivo” measures indirectly detect active thrombin formation. They differ conceptually from the “in vitro” thrombin generation assays under consideration in this review, which directly measure thrombin enzymatic activity.

#### Outcomes

For inclusion, studies must report associated outcomes related to bleeding and/or clinical hemostasis. This will include, but not be limited to, annualized bleed rates (ABRs), bleeding assessment tool scores and/or disease-specific bleeding severity scores, clinical definitions of bleeding as defined by society guidelines, and definitions of effective clinical hemostasis. Studies reporting bleed duration or bleed volume (e.g., following skin punch biopsy among healthy volunteers), surgical studies reporting intraoperative estimated blood loss (EBL), or any other measure of abnormal intraoperative clinical hemostasis will be included. Studies reporting bleeding without using a formal outcome definition as outlined above will still be included. Studies that do not directly report a bleeding or hemostatic outcome among patients or healthy volunteers (e.g., in vitro studies) will be excluded. Studies will be excluded if bleeding, or lack thereof, is exclusively reported as a component of adverse event reporting, provided these studies are not otherwise focused on bleeding/hemostasis as a clinical outcome (e.g., pharmacokinetic or healthy volunteer studies).

#### Context

Studies will be considered if they measured bleeding or clinical hemostasis in a clinical context (e.g., hospital ward, surgical suite or emergency department) or in a clinical research setting (e.g., study of bleeding among healthy volunteers following skin punch biopsy). In addition, studies will be considered irrespective of whether carried out in a community (primary) or academic (tertiary, quaternary) healthcare setting.

#### Study types

We will include randomized controlled trials (RCTs), non-randomized studies (NRS), systematic reviews, and case series. We will consider all publication types, including brief reports, editorials, letters to the editor, and commentaries if they meet eligibility criteria. Narrative literature reviews that do not present original data will be excluded. Systematic reviews will be defined as reviews with a specified review question (i.e., Population, Intervention, Comparator, Outcome; PICO question) that include a systematic search of one or more electronic databases, with clearly defined eligibility criteria, systematic abstract screening, and study selection, data collection by two or more authors and an appraisal of the risk of bias, and synthesis of the included information using either qualitative or quantitative approaches. NRS will include prospective or retrospective cohort studies, case-control studies, non-randomized, quasi-randomized or single-arm interventional trials. Diagnostic test accuracy studies will be excluded if they do not report bleeding/hemostatic outcomes. Conference abstracts and grey literature will be excluded due to the anticipated volume of peer-reviewed literature to be screened. English-language only publications will be considered.

### Information sources and search strategy

The search strategy will be established in collaboration with an information specialist at The Ottawa Hospital (R.S.) with experience in developing search strategies for scoping reviews. We carried out a preliminary literature search using the OVID platform and keywords/MeSH terms related to the topic of interest. The final search strategy will be tailored, based on the preliminary literature search, to balance the need for a comprehensive search strategy and limit the volume of abstracts that could be reasonably screened within project timelines. All team members, including content experts in thrombin generation and coagulation laboratory analyses, hemophilia, anticoagulation reversal and hemostatic therapies, bleeding definitions, and systematic/scoping review methodology, will provide input with respect to the search strategy. Using the OVID platform and our preliminary search results, we will search MEDLINE, EMBASE and the Cochrane Central Register of Controlled Trials (CENTRAL) using a combination of MeSH terms and keywords. Databases will be searched from 1946 to March 21^st^, 2023. The search strategy will use a combination of keywords and MeSH terms detailing the population, concept, and context of interest. Conference abstracts will be removed from Embase and CENTRAL. Grey literature will not be searched. A copy of the search strategy at the time of protocol publication can be found in **S1 Appendix**. The final search strategy will be reviewed by a second library information specialist according to the Peer Review of Electronic Search Strategies (PRESS) checklist [50]. A single reviewer will check reference lists of included studies, including systematic reviews, to identify additional eligible studies not identified through our database search.

### Study selection and data management

Duplicate citations will be removed from the abstract screening list. Covidence^®^ software will be used to maintain a list of titles/abstracts for screening and full-text articles for review. Both title/abstract and full-text screening will be carried out independently and in duplicate by two reviewers (J.R.S. and T.J.) using Covidence^®^. Abstract screening and full-text review will be done by applying the inclusion/exclusion criteria, ensuring that the articles meet the population, concept, and context outlined above. Studies where the methodology/assay used to assess thrombin generation is unclear will be brought forward to full-text review to ensure that the reported method for measurement of thrombin generation falls within the definition outlined above (**Table 4**). One of the study authors will maintain full-text articles in PDF format in a shared folder on a physical hard drive (J.R.S.). A random sample of 50 abstracts will be used to pilot the screening process, ensuring eligibility criteria are consistently applied by both reviewers. Disagreements between reviewers during abstract screening will be resolved by further discussion to reach consensus or consultation with a third party. Disagreements at full-text review will be resolved by discussion or consultation with a third reviewer. Reasons for exclusion at full-text review will be documented in Covidence^®^. The results of abstract screening and full-text review, including reasons for full-text exclusion will be documented and reported using a PRISMA flow diagram [49].

### Data charting

Data abstraction will be carried out independently and in duplicate by two reviewers (J.R.S. and T.J.). Disagreements on abstracted data will be resolved by consensus, or, when needed, through consultation with a third reviewer. A standardized data abstraction form will be developed using Covidence^®^ Data Extraction 2.0. The form will be iteratively modified as reviewers gain familiarity with the content area. All versions of the data abstraction form will be saved, and a summary of the modifications will be documented. The data abstraction form will be piloted using a random sample of 5 full-text articles to ensure consistency in the data abstraction process, and adaptations to the form will be made if needed.

### Data items

Data will be collected on publication/author information, study characteristics, patient population parameters, information on the type of thrombin generation assay used, preanalytical variables, reagent selection and assay conditions, reported TGA parameters, and information on the bleeding/hemostatic outcome used by each study. If applicable, information on comparators for RCT and NRS studies will be recorded. Duration of follow-up will be recorded for longitudinal clinical studies. We will record whether each study reported an association between thrombin generation parameters and bleeding/hemostatic outcomes, and whether formal statistical association/correlation was conducted. If an association between TGA parameters and bleeding/hemostatic outcomes was reported, we will record which TGA parameters were associated and whether the association was statistically significant. Statistical significance will be according to the definition used by each study in question and, where reported, we will record effect estimates and 95% confidence intervals. Given the scoping nature of this review, all outcomes will have equal priority. Reporting of results will be stratified according to healthy volunteer studies versus patient studies (e.g., DOAC-treated patients). Findings from included systematic reviews will be reported according to the synthesized findings from the systematic review in question.

### Data synthesis and reporting

Mapping the information identified will be carried out using Microsoft Excel^®^, Biorender^®^ and other software, as needed, with results presented using qualitative data analytical techniques, including tabulation, graphical representation, and narrative approaches. When presenting results in tabular or graphical form, we will attempt to organize results according to underlying characteristics of interest. These characteristics might include study design, patient population, the thrombin generation assay used, or the reported bleeding/hemostatic outcome. We will seek to identify underlying trends or discrepancies in the data using tabulation. We will consider reporting data using color-coded tabulation to indicate the direction of effect and to facilitate interpretation of the data. Bar/column charts, pie charts, geographic maps, radar plots and bubble charts will also be considered when presenting other aspects of the content area, such as TGA reagent selection or assay conditions, publication/chronological trends, and the geographical distribution of reported TGA use. We will supplement tabulation and graphical representation using narrative synthesis to summarize key findings and knowledge gaps. The approaches outlined above will be tailored to address the study questions and objectives. If needed, we will adapt our approach with increasing familiarity with the content area. Consistent with scoping review methodology [45], we will not undertake a formal critical appraisal of the individual sources of evidence.

## Consultation and stakeholder engagement

To improve the relevance and applicability of our research findings, we will adopt an integrated knowledge translation approach throughout the entire review process. Input on the design of this scoping review was sought from patient partners with lived experience from both the CanVECTOR research network (C.S) and the Canadian Hemophilia Society (D.P.). These patient partners will be involved in all stages of the scoping review process, including interpretation and dissemination of the review findings. During the planning stages of this project, input on review design was also sought from translational scientists who use thrombin generation to study bleeding/hemostasis. This will ensure the findings of this review are relevant to both patients and clinician/translational scientists and stakeholders involved in the research of acquired/congenital bleeding disorders. In addition, we will seek input from relevant Scientific Standardization Committees of the International Society on Thrombosis and Haemostasis on preliminary review findings through stakeholder engagement sessions [23]. Our preliminary findings will be used as a foundation to inform the consultation. The purpose of the consultation/stakeholder engagement session will be to gain additional information, perspectives and enhance the applicability of the scoping review findings [47]. The consultation will also solicit input from international content experts on key review findings, facilitate exchange among stakeholders in the field and inform future research priorities. Important changes to the interpretation of review findings arising from this consultation process, in addition to future research priorities and emerging issues identified by content experts, will be narratively summarized and transparently reported in point form in a dedicated section of the final study report.

## Ethics and dissemination

This scoping review will use published, publicly available information, and as such, research ethics approval will not be required. Strategies for knowledge dissemination will include conference presentations, peer-reviewed publications, and engagement/consultation with international experts and knowledge users as outlined above.

## Implications for research, practice, and policy

Significant progress has been made with thrombin generation assay methodology and implementation over the past two decades. Our understanding of its relation to various disease states and antithrombotic/hemostatic therapies continues to evolve. However, there remains ongoing uncertainty about the utility of thrombin generation as a measure of hemostasis and/or bleeding and its clinical significance. Thrombin generation has proven useful for evaluating the underlying biological mechanisms of hemostasis and thrombosis, but its potential as a surrogate measure of clinical outcomes remains underexploited. Many clinical questions in the field of thrombosis medicine remain unanswered. These questions might be difficult to answer on clinical grounds for a variety of reasons, including challenging participant consent and recruitment in emergencies (e.g., critically ill participants), the need for an unattainably large sample size to achieve sufficient statistical power to answer a clinically meaningful question, or challenges with direct measurement of the outcome of interest (e.g., clinical hemostasis). For a biomarker to achieve widespread acceptance within the scientific and clinical community, it needs a sound scientific basis. It must also demonstrate good accuracy, precision, and correlation with clinically important outcomes. With the introduction of standardized, calibrated, and automated high throughput assays, thrombin generation may have an increasing role in clinical research and the clinical coagulation laboratory.

## Conclusions

Thrombin generation assays have undergone considerable technological and methodological development over the past two decades. Even though these advancements have improved our understanding of its potential clinical utility, its use remains limited to the research laboratory. This scoping review will serve as an important knowledge translation exercise. We will outline knowledge gaps surrounding thrombin generation testing, better delineate its applicability as a clinically relevant assay for bleeding and seek to identify ongoing barriers to its widespread adoption in clinical research, and eventually as a tool for use in the clinical setting.

## Supporting information

S1 Checklist PRISMA-P (Preferred Reporting Items for Systematic review and Meta-Analysis Protocols) 2015 checklist: Recommended items to address in a systematic review protocol*. (DOC)

S1 Appendix Search strategy. (DOCX)

## References

[1] Saba HI, Roberts HR. Hemostasis and Thrombosis: Practical Guidelines in Clinical Management: Wiley; 2014.

[2] Mihalko E, Brown AC. Clot Structure and Implications for Bleeding and Thrombosis. Semin Thromb Hemost. 2020;46(1):96–104. doi: 10.1055/s-0039-1696944 31614389 PMC7460717

[3] Coughlin SR. Protease-activated receptors in hemostasis, thrombosis and vascular biology. J Thromb Haemost. 2005;3(8):1800–14. doi: 10.1111/j.1538-7836.2005.01377.x 16102047

[4] Baglin T. The measurement and application of thrombin generation. Br J Haematol. 2005;130(5):653–61. doi: 10.1111/j.1365-2141.2005.05612.x 16115120

[5] Hemker HC, Béguin S. Phenotyping the clotting system. Thromb Haemost. 2000;84(5):747–51. 11127849

[6] Macfarlane RG, Biggs R. A thrombin generation test; the application in haemophilia and thrombocytopenia. J Clin Pathol. 1953;6(1):3–8. doi: 10.1136/jcp.6.1.3 13034911 PMC1023522

[7] Hemker HC, Wielders S, Kessels H, Béguin S. Continuous registration of thrombin generation in plasma, its use for the determination of the thrombin potential. Thromb Haemost. 1993;70(4):617–24. 7509511

[8] Hemker HC, Giesen PL, Ramjee M, Wagenvoord R, Béguin S. The thrombogram: monitoring thrombin generation in platelet-rich plasma. Thromb Haemost. 2000;83(4):589–91. 10780322

[9] Hemker HC, Giesen P, Al Dieri R, Regnault V, de Smedt E, Wagenvoord R, et al. Calibrated automated thrombin generation measurement in clotting plasma. Pathophysiol Haemost Thromb. 2003;33(1):4–15. doi: 10.1159/000071636 12853707

[10] Depasse F, Binder NB, Mueller J, Wissel T, Schwers S, Germer M, et al. Thrombin generation assays are versatile tools in blood coagulation analysis: A review of technical features, and applications from research to laboratory routine. J Thromb Haemost. 2021;19(12):2907–17. doi: 10.1111/jth.15529 34525255 PMC9291770

[11] Kintigh J, Monagle P, Ignjatovic V. A review of commercially available thrombin generation assays. Res Pract Thromb Haemost. 2018;2(1):42–8. doi: 10.1002/rth2.12048 30046705 PMC6055498

[12] van Veen JJ, Gatt A, Cooper PC, Kitchen S, Bowyer AE, Makris M. Corn trypsin inhibitor in fluorogenic thrombin-generation measurements is only necessary at low tissue factor concentrations and influences the relationship between factor VIII coagulant activity and thrombogram parameters. Blood Coagul Fibrinolysis. 2008;19(3):183–9. doi: 10.1097/MBC.0b013e3282f4bb47 18388496

[13] Douxfils J, Morimont L, Delvigne AS, Devel P, Masereel B, Haguet H, et al. Validation and standardization of the ETP-based activated protein C resistance test for the clinical investigation of steroid contraceptives in women: an unmet clinical and regulatory need. Clin Chem Lab Med. 2020;58(2):294–305. doi: 10.1515/cclm-2019-0471 31444961

[14] Hardy M, Dellanoce C, Douxfils J, Carlo A, Lessire S, Bouvy C, et al. Impact of centrifugation on thrombin generation in healthy subjects and in patients treated with direct oral anticoagulants. Int J Lab Hematol. 2021;43(6):1585–92. doi: 10.1111/ijlh.13655 34252264

[15] Loeffen R, Kleinegris MC, Loubele ST, Pluijmen PH, Fens D, van Oerle R, et al. Preanalytic variables of thrombin generation: towards a standard procedure and validation of the method. J Thromb Haemost. 2012;10(12):2544–54. doi: 10.1111/jth.12012 23020632

[16] Douxfils J, Morimont L, Bouvy C, de Saint-Hubert M, Devalet B, Devroye C, et al. Assessment of the analytical performances and sample stability on ST Genesia system using the STG-DrugScreen application. J Thromb Haemost. 2019;17(8):1273–87. doi: 10.1111/jth.14470 31063645 PMC6852561

[17] Mairesse A, Bayart JL, Desmet S, Lopes Dos Santos H, Saussoy P, Defour JP, et al. Biological variation data and analytical specification goal estimates of the thrombin generation assay with and without thrombomodulin in healthy individuals. Int J Lab Hematol. 2021;43(3):450–7. doi: 10.1111/ijlh.13388 33185328

[18] Wagenvoord RJ, Deinum J, Elg M, Hemker HC. The paradoxical stimulation by a reversible thrombin inhibitor of thrombin generation in plasma measured with thrombinography is caused by alpha-macroglobulin-thrombin. J Thromb Haemost. 2010;8(6):1281–9. doi: 10.1111/j.1538-7836.2010.03822.x 20180821

[19] Dargaud Y, Luddington R, Baglin TP. Elimination of contact factor activation improves measurement of platelet-dependent thrombin generation by calibrated automated thrombography at low-concentration tissue factor. J Thromb Haemost. 2006;4(5):1160–1. doi: 10.1111/j.1538-7836.2006.01905.x 16689781

[20] Dargaud Y, Luddington R, Gray E, Negrier C, Lecompte T, Petros S, et al. Effect of standardization and normalization on imprecision of calibrated automated thrombography: an international multicentre study. Br J Haematol. 2007;139(2):303–9. doi: 10.1111/j.1365-2141.2007.06785.x 17897307

[21] Dargaud Y, Luddington R, Gray E, Lecompte T, Siegemund T, Baglin T, et al. Standardisation of thrombin generation test—which reference plasma for TGT? An international multicentre study. Thromb Res. 2010;125(4):353–6. doi: 10.1016/j.thromres.2009.11.012 19942257

[22] Perrin J, Depasse F, Lecompte T, GEHT F-sCgautao, stated): F-sCgaiFuo, stated F-sCgaiFuo. Large external quality assessment survey on thrombin generation with CAT: further evidence for the usefulness of normalisation with an external reference plasma. Thromb Res. 2015;136(1):125–30. 25563679 10.1016/j.thromres.2014.12.015

[23] Ninivaggi M, de Laat-Kremers R, Tripodi A, Wahl D, Zuily S, Dargaud Y, et al. Recommendations for the measurement of thrombin generation: Communication from the ISTH SSC Subcommittee on Lupus Anticoagulant/Antiphospholipid Antibodies. J Thromb Haemost. 2021;19(5):1372–8. doi: 10.1111/jth.15287 33880866

[24] Dargaud Y, Wolberg AS, Gray E, Negrier C, Hemker HC, Subcommittee on Factor VIII FcI, and Rare Coagulation Disorders. Proposal for standardized preanalytical and analytical conditions for measuring thrombin generation in hemophilia: communication from the SSC of the ISTH. J Thromb Haemost. 2017;15(8):1704–7. 28656617 10.1111/jth.13743 PMC5680042

[25] Shaw JR, Castellucci LA, Siegal D, Carrier M. DOAC-associated bleeding, hemostatic strategies, and thrombin generation assays—a review of the literature. J Thromb Haemost. 2022. doi: 10.1016/j.jtha.2022.11.029 36696204

[26] Ninivaggi M, de Laat-Kremers RMW, Carlo A, de Laat B. ST Genesia reference values of 117 healthy donors measured with STG-BleedScreen, STG-DrugScreen and STG-ThromboScreen reagents. Res Pract Thromb Haemost. 2021;5(1):187–96. doi: 10.1002/rth2.12455 33537543 PMC7845068

[27] Hemker HC, Giesen P, AlDieri R, Regnault V, de Smed E, Wagenvoord R, et al. The calibrated automated thrombogram (CAT): a universal routine test for hyper- and hypocoagulability. Pathophysiol Haemost Thromb. 2002;32(5–6):249–53. doi: 10.1159/000073575 13679651

[28] Samuelson BT, Cuker A, Siegal DM, Crowther M, Garcia DA. Laboratory Assessment of the Anticoagulant Activity of Direct Oral Anticoagulants: A Systematic Review. Chest. 2017;151(1):127–38. doi: 10.1016/j.chest.2016.08.1462 27637548 PMC5310120

[29] Douxfils J, Ageno W, Samama CM, Lessire S, Ten Cate H, Verhamme P, et al. Laboratory testing in patients treated with direct oral anticoagulants: a practical guide for clinicians. J Thromb Haemost. 2018;16(2):209–19. doi: 10.1111/jth.13912 29193737

[30] Tripodi A. Thrombin generation: a global coagulation procedure to investigate hypo- and hyper-coagulability. Haematologica. 2020;105(9):2196–9. doi: 10.3324/haematol.2020.253047 33054044 PMC7556623

[31] Tripodi A, Mannucci PM. The coagulopathy of chronic liver disease. N Engl J Med. 2011;365(2):147–56. doi: 10.1056/NEJMra1011170 21751907

[32] Lebreton A, Sinegre T, Lecompte T, Talon L, Abergel A, Lisman T. Thrombin Generation and Cirrhosis: State of the Art and Perspectives. Semin Thromb Hemost. 2020;46(6):693–703. doi: 10.1055/s-0040-1715102 32820480

[33] Santagostino E, Mancuso ME, Tripodi A, Chantarangkul V, Clerici M, Garagiola I, et al. Severe hemophilia with mild bleeding phenotype: molecular characterization and global coagulation profile. J Thromb Haemost. 2010;8(4):737–43. doi: 10.1111/j.1538-7836.2010.03767.x 20102490

[34] Zekavat OR, Haghpanah S, Dehghani J, Afrasiabi A, Peyvandi F, Karimi M. Comparison of thrombin generation assay with conventional coagulation tests in evaluation of bleeding risk in patients with rare bleeding disorders. Clin Appl Thromb Hemost. 2014;20(6):637–44. doi: 10.1177/1076029613475473 23393288

[35] Váradi K, Negrier C, Berntorp E, Astermark J, Bordet JC, Morfini M, et al. Monitoring the bioavailability of FEIBA with a thrombin generation assay. J Thromb Haemost. 2003;1(11):2374–80. doi: 10.1046/j.1538-7836.2003.00450.x 14629472

[36] Hoffman M, Dargaud Y. Mechanisms and monitoring of bypassing agent therapy. J Thromb Haemost. 2012;10(8):1478–85. doi: 10.1111/j.1538-7836.2012.04793.x 22632160

[37] Shaw JR, Siegal DM. Prothrombin complex concentrates for DOAC-associated bleeding, global coagulation assays, and assessments of clinical hemostasis: How to gauge the impact? Res Pract Thromb Haemost. 2020;4(5):677–9. doi: 10.1002/rth2.12367 32685875 PMC7354417

[38] Dargaud Y, Negrier C, Rusen L, Windyga J, Georgiev P, Bichler J, et al. Individual thrombin generation and spontaneous bleeding rate during personalized prophylaxis with Nuwiq. Haemophilia. 2018;24(4):619–27. 29855112 10.1111/hae.13493

[39] Khorsand N, Beyer-Westendorf J, Sarode R, Schulman S, Meijer K. Definition of haemostatic effectiveness in interventions used to treat major bleeding: Communication from the ISTH SSC Subcommittee on Control of Anticoagulation. J Thromb Haemost. 2021;19(4):1112–5. doi: 10.1111/jth.15222 33792175

[40] Dargaud Y, Lienhart A, Negrier C. Prospective assessment of thrombin generation test for dose monitoring of bypassing therapy in hemophilia patients with inhibitors undergoing elective surgery. Blood. 2010;116(25):5734–7. doi: 10.1182/blood-2010-06-291906 20810929

[41] Dargaud Y, Lienhart A, Janbain M, Le Quellec S, Enjolras N, Negrier C. Use of thrombin generation assay to personalize treatment of breakthrough bleeds in a patient with hemophilia and inhibitors receiving prophylaxis with emicizumab. Haematologica. 2018;103(4):e181–e3. doi: 10.3324/haematol.2017.185330 29472355 PMC5865419

[42] Coakley M, Hall JE, Evans C, Duff E, Billing V, Yang L, et al. Assessment of thrombin generation measured before and after cardiopulmonary bypass surgery and its association with postoperative bleeding. J Thromb Haemost. 2011;9(2):282–92. doi: 10.1111/j.1538-7836.2010.04146.x 21091865

[43] Zalewski J, Stepien K, Nowak K, Caus S, Butenas S, Undas A. Delayed Thrombin Generation Is Associated with Minor Bleedings in Venous Thromboembolism Patients on Rivaroxaban: Usefulness of Calibrated Automated Thrombography. J Clin Med. 2020;9(7). doi: 10.3390/jcm9072018 32605001 PMC7409038

[44] Bloemen S, Zwaveling S, Ten Cate H, Ten Cate-Hoek A, de Laat B. Prediction of bleeding risk in patients taking vitamin K antagonists using thrombin generation testing. PLoS One. 2017;12(5):e0176967. doi: 10.1371/journal.pone.0176967 28472104 PMC5417600

[45] Peters MD, Godfrey CM, Khalil H, McInerney P, Parker D, Soares CB. Guidance for conducting systematic scoping reviews. Int J Evid Based Healthc. 2015;13(3):141–6. doi: 10.1097/XEB.0000000000000050 26134548

[46] Arksey H, O’Malley L. Scoping studies: towards a methodological framework. International Journal of Social Research Methodology. 2005;8(1):19–32.

[47] Levac D, Colquhoun H, O’Brien KK. Scoping studies: advancing the methodology. Implement Sci. 2010;5:69. doi: 10.1186/1748-5908-5-69 20854677 PMC2954944

[48] Peters M, Godfrey C, McInerney P, Munn Z, Tricco A, Khalil H. Chapter 11: Scoping Reviews (2020 version). In: Aromataris E, Munn Z (Editors). JBI Manual for Evidence Synthesis, JBI, 2020. Available from https://synthesismanual.jbi.global. 10.46658/JBIMES-20-12.

[49] Tricco AC, Lillie E, Zarin W, O’Brien KK, Colquhoun H, Levac D, et al. PRISMA Extension for Scoping Reviews (PRISMA-ScR): Checklist and Explanation. Ann Intern Med. 2018;169(7):467–73. doi: 10.7326/M18-0850 30178033

[50] McGowan J, Sampson M, Salzwedel DM, Cogo E, Foerster V, Lefebvre C. PRESS Peer Review of Electronic Search Strategies: 2015 Guideline Statement. J Clin Epidemiol. 2016;75:40–6. 27005575 10.1016/j.jclinepi.2016.01.021

