# Supplementary material for: Thrombin generation, bleeding and hemostasis in humans: Protocol for a scoping review of the literature
Source: PLoS One. 2023 Nov 1;18(11):e0293632. doi: 10.1371/journal.pone.0293632 (PMC10619830; doi:10.1371/journal.pone.0293632)
Supplement: S1 Appendix — (DOCX) [file pone.0293632.s002.docx]

**S1 Appendix.** Search Strategy

Ovid MEDLINE(R) ALL <1946 to March 20, 2023>

1 ((thrombin adj3 generation) and (assay* or test* or measure*)).tw,kf. 3815

2 (thrombin generation and laboratory).tw,kf. 460

3 (thrombograph* or thrombinograph*).mp. or tga.ti,kf. 1195

4 (calibrated automated Thromb* or thrombinoscop*).tw,kf. 565

5 st genesia.tw,kf. 39

6 (innovance adj3 etp).tw,kf. 5

7 (innovance and thrombin).tw,kf. 9

8 technothrombin.tw,kf. 18

9 ceveron alpha*.tw,kf. 6

10 endogenous thrombin potential*.tw,kf. 918

11 (revers* adj10 thrombin generation).tw,kf. 67

12 or/1-11 5210

13 exp Blood Coagulation Factors/ai [Antagonists & Inhibitors] 14774

14 prothrombin complex concentrate*.tw,kf. 2116

15 pcc.tw,kf. 12567

16 bebulin.tw,kf. 16

17 octaplex.tw,kf. 41

18 apcc.tw,kf. 342

19 feiba.tw,kf. 324

20 Factor VIIa/ 4116

21 (recombinant FVIIa or rFVIIa or novosen or sevenfact).tw,kf. 1878

22 fresh frozen plasma.tw,kf. 6659

23 ffp.tw,kf. 3042

24 (andexanet or andexxa).tw,kf. 339

25 idarucizumab.mp. 586

26 praxbind.tw,kf. 34

27 ciraparantag.mp. 63

28 aripazine.mp. 25

29 (antifibrinolytic agent* or aminocaproic acid* or tranexamic acid*).mp. 15420

30 fibrinogen concentrate*.tw,kf. 634

31 (riastap or fibryga or clottafact).mp. 35

32 Factor VII concentrat*.mp. 103

33 (PD FVII or plasma derived FVII).tw,kf. 15

34 Factor VII/tu [Therapeutic Use] 703

35 Factor VIII/ 17411

36 advate.mp. 121

37 hemofil m.mp. 30

38 kogenate fs.mp. 32

39 koate.mp. 20

40 kovaltry.mp. 26

41 novoeight.mp. 22

42 nuwiq.mp. 25

43 recombinate.mp. 110

44 xyntha.mp. 13

45 adynovate.mp. 14

46 afstyla.mp. 10

47 eloctate.mp. 25

48 esperoct.mp. 11

49 jivi.mp. 11

50 factane.mp. 6

51 octanate.mp. 23

52 (Efanesoctocog alfa or Altuviiio).mp. 4

53 recombinant porcine sequence.mp. 8

54 obizur.mp. 18

55 Factor IX/tu [Therapeutic Use] 1110

56 (obizur or alphanine or benefix or ixinity or mononine or rixubis or alprolix or idelvion or rebinyn).mp. 129

57 coagadex.mp. 2

58 Factor X/tu [Therapeutic Use] 113

59 Factor XI/tu [Therapeutic Use] 47

60 (pd fxI or Hemoleven).mp. 7

61 Factor XIII/tu [Therapeutic Use] 489

62 (corifact or tretten).mp. 7

63 bispecific monoclonal antibod*.tw,kf. 397

64 (emicizumab or ace910 or hemlibra).mp. 510

65 (Anti-tissue factor pathway inhibitor* or anti tfpi agent* or anti tfpi inhibitor*).tw,kf. 28

66 concizumab.mp. 49

67 marstacimab.mp. 11

68 RNA, Small Interfering/tu [Therapeutic Use] 2131

69 fitusiran.mp. 34

70 aptamer*.tw,kf. 17601

71 bt200.mp. 12

72 von Willebrand Factor/tu [Therapeutic Use] 412

73 (humate p or alphanate or wilate or voncento).mp. 105

74 recombinant vwf.mp. 207

75 recombinant von willebrand factor.tw,kf. 100

76 vonvendi.mp. 10

77 or/13-76 91693

78 exp anticoagulants/ 241437

79 (warfarin or acenocouramol or phenprocoumon or fluindione).mp. 34739

80 vitamin k antagonist*.tw,kf. 7875

81 Vitamin K/ai [Antagonists & Inhibitors] 3089

82 Factor Xa Inhibitors/ 6005

83 (apixaban or betrixaban or edoxaban or rivaroxaban).mp. 10478

84 Antithrombins/ 6869

85 (antithrombin* or thrombin inhibitor*).tw,kf. 21399

86 (dabigatran or melagatran* or argatroban or bivalirudin).mp. 10026

87 (low molecular weight heparin* or unfractionated heparin* or lmwh).tw,kf. 18275

88 (dalteparin or enoxaparin or nadroparin or semuloparin or tinzaparin).mp. 8246

89 fondaparinux.mp. 2174

90 (heparinoid* or danaparoid).mp. 1967

91 (ionis fxI or osocimab or abelacimab or milvexian or xisomab or fesomersen or asundexian).mp. 36

92 Platelet Aggregation Inhibitors/ 40687

93 (acetylsalicylic acid* or aspirin or clopidogrel or prasugrel or ticagrelor or ticlopidine or cangrelor or abciximab or dipyridamole).mp. 97060

94 (antiplatelet adj2 (agent* or drug* or inhibitor*)).tw,kf. 10894

95 or/78-94 363097

96 blood coagulation disorders, inherited/ or hemophilia a/ or hemophilia b/ or exp von willebrand diseases/ 29984

97 h?emophil*.tw,kf. 52866

98 von willebrand.tw,kf. 20104

99 (Afibrinogenemia or dysfibrinogenemia).mp. 2848

100 blood platelet disorders/ or bernard-soulier syndrome/ or gray platelet syndrome/ or exp platelet storage pool deficiency/ 5131

101 (bernard soulier or gray platelet syndrome* or storage pool disorder*).tw,kf. 902

102 May-Hegglin Anomaly.mp. 233

103 hermanski-pudlak syndrome/ or wiskott-aldrich syndrome/ 2002

104 (wiskott aldrich syndrome or chediak higashi or hermanski-pudlak).tw,kf. 3413

105 glanzmann.tw,kf. 631

106 Thrombasthenia/ 999

107 Thrombasthenia.tw,kf. 1400

108 ((rare or inherited) adj2 (bleed* or coagulat*) adj2 (disorder* or deficienc*)).tw,kf. 2093

109 Blood Coagulation Disorders/ 16668

110 acquired h?emophil*.tw,kf. 1319

111 consumptive coagulopathy.mp. 684

112 Disseminated Intravascular Coagulation/ 11584

113 Disseminated Intravascular Coagulation.tw,kf. 11206

114 liver diseases/ or exp liver cirrhosis/ or Liver Failure, Acute/ 172270

115 ((liver or hepatic) adj10 cirrho*).tw,kf. or cirrhosis.ti. 85465

116 Blood Platelet Disorders/ 4088

117 (Acquired disorder* of platelet function or acquired platelet disorder* or acquired platelet dysfunction*).tw,kf. 128

118 immune thrombocytopenia.mp. or exp Purpura, Thrombocytopenic, Idiopathic/ 9865

119 platelet dysfunction.mp. 1863

120 Thrombocytopenia/ 30088

121 thrombocytopenia.tw,kf. 57529

122 iatrogenic bleeding.mp. or Postoperative Hemorrhage/ 11099

123 ((postoper* or surg* or postsurg*) adj2 (bleed* or h?emorrhag*)).tw,kf. 16327

124 ((postoper* or surg* or postsurg*) and (bleed* or h?emorrhag*)).ti. 7657

125 punch biops*.tw,kf. 4046

126 or/96-125 414683

127 77 or 95 or 126 812295

128 12 and 127 3069

129 exp animals/ not humans/ 5104362

130 128 not 129 2891

131 limit 130 to english language 2811

Embase Classic+Embase <1947 to 2023 March 20>

1 ((thrombin adj3 generation) and (assay* or test* or measure*)).tw. 8525

2 (thrombin generation and laboratory).tw. 1083

3 (thrombograph* or thrombinograph*).tw. or tga.ti. 1297

4 (calibrated automated Thromb* or thrombinoscop*).tw. 1841

5 st genesia.tw. 117

6 (innovance adj3 etp).tw. 12

7 (innovance and thrombin).tw. 55

8 technothrombin.tw. 118

9 ceveron alpha*.tw. 66

10 endogenous thrombin potential*.tw. 2335

11 (revers* adj10 thrombin generation).tw. 149

12 or/1-11 10180

13 blood clotting factor/ or exp *blood clotting factor/ 115597

14 prothrombin complex/ 5728

15 prothrombin complex concentrate.tw. 2784

16 pcc.tw. 16864

17 bebulin.mp. or blood clotting factor 9 complex/ 465

18 octaplex.mp. 299

19 apcc.tw. 1007

20 feiba.mp. or activated prothrombin complex/ 2849

21 blood clotting factor 7a/ 3464

22 (recombinant FVIIa or rFVIIa or novosen or sevenfact).tw. 3770

23 fresh frozen plasma/ 21333

24 fresh frozen plasma.tw. 11729

25 ffp.tw. 7248

26 *andexanet alfa/ 332

27 (andexanet or andexxa).tw. 612

28 *idarucizumab/ 504

29 idarucizumab.tw. 869

30 praxbind.tw. 166

31 *ciraparantag/ 37

32 ciraparantag.tw. 75

33 aripazine.tw. 36

34 *antifibrinolytic agent/ 2458

35 *aminocaproic acid/ 2976

36 *tranexamic acid/ 6481

37 (antifibrinolytic agent* or aminocaproic acid* or tranexamic acid*).tw. 13301

38 *fibrinogen concentrate/ 451

39 fibrinogen concentrate*.tw. 1287

40 (riastap or fibryga or clottafact).tw. 205

41 Factor VII concentrat*.tw. 143

42 (PD FVII or plasma derived FVII).tw. 46

43 blood clotting factor 7/ 11754

44 *recombinant blood clotting factor 8/ 2327

45 advate.tw. 962

46 *blood clotting factor 8 concentrate/ 1151

47 hemofil.tw. 225

48 kogenate.tw. 768

49 koate.tw. 186

50 kovaltry.tw. 143

51 novoeight.tw. 148

52 nuwiq.tw. 136

53 recombinate.tw. 447

54 xyntha.tw. 145

55 adynovate.tw. 131

56 afstyla.tw. 82

57 eloctate.tw. 179

58 jivi.tw. 85

59 factane.tw. 56

60 octanate.tw. 143

61 (Efanesoctocog alfa or Altuviiio).tw. 18

62 recombinant porcine sequence.tw. 39

63 obizur.tw. 80

64 *blood clotting factor 9/ 3442

65 (obizur or alphanine or benefix or ixinity or mononine or rixubis or alprolix or idelvion or rebinyn).tw. 924

66 *blood clotting factor 10 concentrate/ 27

67 coagadex.tw. 16

68 *blood clotting factor 11/ 1290

69 (pd fxI or Hemoleven).tw. 42

70 *blood clotting factor 13/ 2292

71 *blood clotting factor 13 concentrate/ 102

72 (corifact or tretten).tw. 40

73 bispecific monoclonal antibod*.tw. 584

74 (emicizumab or ace910 or hemlibra).tw. 1457

75 *emicizumab/ 990

76 *tissue factor pathway inhibitor/ 1445

77 (Anti-tissue factor pathway inhibitor* or anti tfpi agent* or anti tfpi inhibitor*).tw. 76

78 *concizumab/ 85

79 concizumab.tw. 112

80 *marstacimab/ 28

81 marstacimab.tw. 19

82 exp *small interfering RNA/ 17316

83 fitusiran.tw. 98

84 *aptamer/ 8130

85 aptamer*.tw. 19042

86 bt200.tw. 25

87 *von Willebrand factor/ 9338

88 *blood clotting factor 8 concentrate/ 1151

89 (humate p or alphanate or wilate or voncento).tw. 661

90 recombinant vwf.tw. 424

91 recombinant von willebrand factor.tw. 185

92 *recombinant von Willebrand factor/ 125

93 vonvendi.tw. 51

94 or/13-93 218898

95 exp anticoagulant agent/ 814102

96 (warfarin or acenocouramol or phenprocoumon or fluindione).tw. 47424

97 vitamin k antagonist*.tw. 13345

98 exp *blood clotting factor 10a inhibitor/ 29219

99 (apixaban or betrixaban or edoxaban or rivaroxaban).tw. 19676

100 *antithrombin/ 3317

101 (antithrombin* or thrombin inhibitor*).tw. 29849

102 exp *thrombin inhibitor/ 19899

103 (dabigatran or melagatran* or argatroban or bivalirudin).tw. 16635

104 exp *low molecular weight heparin/ 17381

105 (low molecular weight heparin* or unfractionated heparin* or lmwh).tw. 30872

106 (dalteparin or enoxaparin or nadroparin or semuloparin or tinzaparin).tw. 12823

107 fondaparinux.tw. 3301

108 (heparinoid* or danaparoid).tw. 2109

109 exp *blood clotting factor 11a inhibitor/ 140

110 (ionis fxI or osocimab or abelacimab or milvexian or xisomab or fesomersen or asundexian).tw. 74

111 (acetylsalicylic acid* or aspirin or clopidogrel or prasugrel or ticagrelor or ticlopidine or cangrelor or abciximab or dipyridamole).tw. 167515

112 *antithrombocytic agent/ 13372

113 (antiplatelet adj2 (agent* or drug* or inhibitor*)).tw. 17207

114 or/95-113 833033

115 exp *blood clotting disorder/ 401069

116 h?emophil*.tw. 77394

117 von willebrand.tw. 30275

118 (Afibrinogenemia or dysfibrinogenemia).tw. 1619

119 exp *thrombocyte disorder/ 71056

120 (bernard soulier or gray platelet syndrome* or storage pool disorder*).tw. 1373

121 may hegglin anomal*.tw. 304

122 *Wiskott Aldrich syndrome/ 1593

123 (wiskott aldrich syndrome or chediak higashi or hermanski-pudlak).tw. 4802

124 glanzmann.tw. 1163

125 *Glanzmann disease/ 1436

126 Thrombasthenia.tw. 2181

127 ((rare or inherited) adj2 (bleed* or coagulat*) adj2 (disorder* or deficienc*)).tw. 4378

128 *blood clotting disorder/ 12603

129 *disseminated intravascular clotting/ 10412

130 Disseminated Intravascular Coagulation.tw. 15182

131 consumptive coagulopathy.tw. 1091

132 exp *liver cirrhosis/ or *liver disease/ 142133

133 *acute liver failure/ 4599

134 ((liver or hepatic) adj10 cirrho*).tw. or cirrhosis.ti. 133723

135 (Acquired disorder* of platelet function or acquired platelet disorder* or acquired platelet dysfunction*).tw. 190

136 *autoimmune thrombocytopenia/ or *idiopathic thrombocytopenic purpura/ 10668

137 immune thrombocytopenia.tw. 8352

138 *thrombocytopenia/ 25819

139 Thrombocytopenia.tw. 103582

140 *postoperative hemorrhage/ 5962

141 iatrogenic bleeding.tw. 128

142 ((postoper* or surg* or postsurg*) adj2 (bleed* or h?emorrhag*)).tw. 25166

143 ((postoper* or surg* or postsurg*) and (bleed* or h?emorrhag*)).ti. 9815

144 punch biops*.tw. 7931

145 *punch biopsy/ 739

146 or/115-145 780860

147 94 or 114 or 146 1601617

148 12 and 147 8476

149 conference abstract.pt. 4705079

150 (exp animals/ or animal experiment/ or nonhumans/) not exp humans/ 6252011

151 149 or 150 10518759

152 148 not 151 3756

153 limit 152 to english language 3619

EBM Reviews - Cochrane Central Register of Controlled Trials <February 2023>

1 ((thrombin adj3 generation) and (assay* or test* or measure*)).tw,kw. 507

2 (thrombin generation and laboratory).tw,kw. 54

3 (thrombograph* or thrombinograph*).mp. or tga.ti,kw. 36

4 (calibrated automated Thromb* or thrombinoscop*).tw,kw. 53

5 st genesia.tw,kw. 2

6 (innovance adj3 etp).tw,kw. 0

7 (innovance and thrombin).tw,kw. 2

8 technothrombin.tw,kw. 1

9 ceveron alpha*.tw,kw. 0

10 endogenous thrombin potential*.tw,kw. 155

11 (revers* adj10 thrombin generation).tw,kw. 17

12 or/1-11 627

13 exp Blood Coagulation Factors/ai [Antagonists & Inhibitors] 15

14 prothrombin complex concentrate*.tw,kf. 228

15 pcc.tw,kf. 618

16 bebulin.tw,kf. 3

17 octaplex.tw,kf. 18

18 apcc.tw,kf. 39

19 feiba.tw,kf. 60

20 Factor VIIa/ 183

21 (recombinant FVIIa or rFVIIa or novosen or sevenfact).tw,kf. 265

22 fresh frozen plasma.tw,kf. 781

23 ffp.tw,kf. 554

24 (andexanet or andexxa).tw,kf. 34

25 idarucizumab.mp. 35

26 praxbind.tw,kf. 2

27 ciraparantag.mp. 12

28 aripazine.mp. 0

29 (antifibrinolytic agent* or aminocaproic acid* or tranexamic acid*).mp. 3927

30 fibrinogen concentrate*.tw,kf. 228

31 (riastap or fibryga or clottafact).mp. 38

32 Factor VII concentrat*.mp. 16

33 (PD FVII or plasma derived FVII).tw,kf. 3

34 Factor VII/tu [Therapeutic Use] 12

35 Factor VIII/ 426

36 advate.mp. 61

37 hemofil m.mp. 10

38 kogenate fs.mp. 9

39 koate.mp. 2

40 kovaltry.mp. 6

41 novoeight.mp. 7

42 nuwiq.mp. 7

43 recombinate.mp. 8

44 xyntha.mp. 6

45 adynovate.mp. 4

46 afstyla.mp. 2

47 eloctate.mp. 9

48 esperoct.mp. 0

49 jivi.mp. 11

50 factane.mp. 3

51 octanate.mp. 6

52 (Efanesoctocog alfa or Altuviiio).mp. 0

53 recombinant porcine sequence.mp. 0

54 obizur.mp. 0

55 Factor IX/tu [Therapeutic Use] 0

56 (obizur or alphanine or benefix or ixinity or mononine or rixubis or alprolix or idelvion or rebinyn).mp. 25

57 coagadex.mp. 0

58 Factor X/tu [Therapeutic Use] 0

59 Factor XI/tu [Therapeutic Use] 0

60 (pd fxI or Hemoleven).mp. 0

61 Factor XIII/tu [Therapeutic Use] 17

62 (corifact or tretten).mp. 0

63 bispecific monoclonal antibod*.tw,kf. 23

64 (emicizumab or ace910 or hemlibra).mp. 82

65 (Anti-tissue factor pathway inhibitor* or anti tfpi agent* or anti tfpi inhibitor*).tw,kf. 13

66 concizumab.mp. 34

67 marstacimab.mp. 6

68 RNA, Small Interfering/tu [Therapeutic Use] 1

69 fitusiran.mp. 18

70 aptamer*.tw,kf. 93

71 bt200.mp. 6

72 von Willebrand Factor/tu [Therapeutic Use] 0

73 (humate p or alphanate or wilate or voncento).mp. 30

74 recombinant vwf.mp. 7

75 recombinant von willebrand factor.tw,kf. 12

76 vonvendi.mp. 4

77 or/13-76 6820

78 exp anticoagulants/ 13697

79 (warfarin or acenocouramol or phenprocoumon or fluindione).mp. 5491

80 vitamin k antagonist*.tw,kf. 1130

81 Vitamin K/ai [Antagonists & Inhibitors] 0

82 Factor Xa Inhibitors/ 726

83 (apixaban or betrixaban or edoxaban or rivaroxaban).mp. 3501

84 Antithrombins/ 437

85 (antithrombin* or thrombin inhibitor*).tw,kf. 2164

86 (dabigatran or melagatran* or argatroban or bivalirudin).mp. 1914

87 (low molecular weight heparin* or unfractionated heparin* or lmwh).tw,kf. 4692

88 (dalteparin or enoxaparin or nadroparin or semuloparin or tinzaparin).mp. 3669

89 fondaparinux.mp. 450

90 (heparinoid* or danaparoid).mp. 234

91 (ionis fxI or osocimab or abelacimab or milvexian or xisomab or fesomersen or asundexian).mp. 37

92 Platelet Aggregation Inhibitors/ 5008

93 (acetylsalicylic acid* or aspirin or clopidogrel or prasugrel or ticagrelor or ticlopidine or cangrelor or abciximab or dipyridamole).mp. 22301

94 (antiplatelet adj2 (agent* or drug* or inhibitor*)).tw,kf. 1678

95 or/78-94 42711

96 blood coagulation disorders, inherited/ or hemophilia a/ or hemophilia b/ or exp von willebrand diseases/ 594

97 h?emophil*.tw,kf. 3514

98 von willebrand.tw,kf. 1278

99 (Afibrinogenemia or dysfibrinogenemia).mp. 33

100 blood platelet disorders/ or bernard-soulier syndrome/ or gray platelet syndrome/ or exp platelet storage pool deficiency/ 36

101 (bernard soulier or gray platelet syndrome* or storage pool disorder*).tw,kf. 1

102 May-Hegglin Anomaly.mp. 0

103 hermanski-pudlak syndrome/ or wiskott-aldrich syndrome/ 11

104 (wiskott aldrich syndrome or chediak higashi or hermanski-pudlak).tw,kf. 17

105 glanzmann.tw,kf. 12

106 Thrombasthenia/ 4

107 Thrombasthenia.tw,kf. 9

108 ((rare or inherited) adj2 (bleed* or coagulat*) adj2 (disorder* or deficienc*)).tw,kf. 54

109 Blood Coagulation Disorders/ 437

110 acquired h?emophil*.tw,kf. 16

111 consumptive coagulopathy.mp. 11

112 Disseminated Intravascular Coagulation/ 124

113 Disseminated Intravascular Coagulation.tw,kf. 331

114 liver diseases/ or exp liver cirrhosis/ or Liver Failure, Acute/ 4916

115 ((liver or hepatic) adj10 cirrho*).tw,kf. or cirrhosis.ti. 6863

116 Blood Platelet Disorders/ 31

117 (Acquired disorder* of platelet function or acquired platelet disorder* or acquired platelet dysfunction*).tw,kf. 9

118 immune thrombocytopenia.mp. or exp Purpura, Thrombocytopenic, Idiopathic/ 742

119 platelet dysfunction.mp. 178

120 Thrombocytopenia/ 1150

121 thrombocytopenia.tw,kf. 6937

122 iatrogenic bleeding.mp. or Postoperative Hemorrhage/ 1506

123 ((postoper* or surg* or postsurg*) adj2 (bleed* or h?emorrhag*)).tw,kf. 3657

124 ((postoper* or surg* or postsurg*) and (bleed* or h?emorrhag*)).ti. 1295

125 punch biops*.tw,kf. 484

126 or/96-125 28180

127 77 or 95 or 126 73012

128 12 and 127 498

129 limit 128 to (conference proceeding or trial registry record) 186

130 128 not 129 312

131 limit 130 to english language 308
